# Khat use and related determinants among pregnant women within Haramaya, Ethiopia: a mixed methods study

**DOI:** 10.3389/fgwh.2024.1359689

**Published:** 2024-05-09

**Authors:** Elizabeth A. Wood, Heather Stark, Stuart J. Case, Barbara Sousa, Melanie Moreno, Aboma Motuma, Tara Wilfong

**Affiliations:** ^1^ Eck Institute for Global Health, University of Notre Dame, Notre Dame, IN, United States; ^2^ College of Public Health & Health Professions, University of Florida, Gainesville, FL, United States; ^3^ School of Nursing and Midwifery, College of Health and Medical Sciences, Haramaya University, Harar, Ethiopia; ^4^ School of Public Health, College of Health and Medical Sciences, Haramaya University, Harar, Ethiopia

**Keywords:** pregnant women, Khat, Ethiopia, mixed methods, pesticides

## Abstract

**Introduction:**

Khat, a green leafy plant grown in East Africa and throughout the Arabian Peninsula, is chewed for its psychoactive and amphetamine-like effects, serving as a significant aspect of culture, economic livelihood, and global trade. Khat consumption during pregnancy has been associated with adverse effects, including anemia, premature rupture of membranes, and low birth weight, among others.

**Methods:**

This cross-sectional, explanatory sequential mixed methods study was conducted in the Haramaya District of eastern Ethiopia using a questionnaire and focus group discussions. Questionnaires assessed socio-demographic information, pregnancy history, and diet, including khat use. Data were analyzed using SPSS v28 to include descriptive statistics, proportions, odds ratios, binary logistic regression, and chi-square analysis. FGDs expanded on the knowledge, attitudes, and practices of khat in the region, including pregnant or lactating women from two different kebeles. Two independent reviewers conducted a qualitative content analysis to examine the qualitative findings from the FGDs. Transcripts from the focus groups were entered into NVivo 14 to aid in capturing salient themes.

**Results:**

A total of 444 pregnant women with a median age of 25 years completed the questionnaire. Two-thirds of the women, 66.9%, reported currently consuming khat while pregnant, and 72.7% of them reported daily consumption. The FGD analysis resulted in the discovery of five themes: Economic Livelihood, Maternal Significance, Medicinal Implications of Khat, Pesticide Use, and Social and Cultural Applications.

**Discussion:**

This study revealed an alarming high prevalence of khat consumption among pregnant women in the Haramaya District, highlighting the pressing need for long-term studies to assess the health consequences. The role of khat as both an economic staple and an energy source for daily activities underscores the challenges in curbing its use. The documented health risks associated with the chemicals used in khat cultivation, including cancer, call for interventions to enhance safe agricultural practices in households involved in khat farming.

## Introduction

Khat is a green leafy plant cultivated in East Africa, whose leaves are chewed and consumed for its euphoric effect, having psychoactive and amphetamine-like properties (1). In eastern Ethiopia, khat chewing is an integral part of traditional and social norms and a significant contributor to economic livelihood, as it is the leading cash crop in the area (2), being sold locally and exported internationally (3). Khat is primarily found in certain countries of East Africa and the Republic of Yemen, with the Oromia region of eastern Ethiopia being a significant source of khat growing, chewing, and distribution globally (4). According to Haile and Lakew (5), the prevalence of khat chewing was highest in the Harari Region of Ethiopia, with other areas such as Dire Dawa also among the highest concentrated areas. Khat chewing practices originated in the 13th century, with global distribution increasing recently despite being illegal in the European Union and the United States (1). It is used primarily for its stimulant properties, having an addictive nature due to the small amount of cathinone ingested when chewed (6). Studies have noted various reasons for chewing khat, including increasing efficiency and energy during work (7), maintaining student alertness during school, and using it in social gatherings and religious settings (8). Khat consumption is associated with psychosis, irritability, anorexia, hypertension, increased gastrointestinal disorders, and possible insomnia (4, 9). In Haramaya and other areas of Ethiopia, khat is a significant crop that plays a vital role in the livelihoods of people. A large portion of the adult and economically active population is engaged in farming, with 35% of the population working as farmers (10). It is a significant source of income as it is exported to Djibouti and Somalia and traded in Harar (11). Besides khat, other vegetables and fruits are important cash crops in the area, with maize and sorghum also commonly grown alongside khat (12). The cultivation and trade of khat have significant socioeconomic and cultural impacts on households in the region. It is not only a source of income but also an integral part of social events and gatherings. The use of pesticides, particularly Dichlorodiphenyltrichloroethane (DDT) and malathion, is a prevalent practice among khat farmers in the Chiro Woreda region, as they strive to combat pests, insects, and diseases that significantly impair yield (13). Investigations into khat and water samples within Haramaya revealed the presence of various pesticides, including aldrin, dialdrin, BHC, diazinon, DDT, 4,4-DDE, and heptachlor, with diazinon and DDT levels exceeding the maximum residue limit in certain khat samples (14). Moreover, a survey in Haramaya highlighted that farmers employ pesticides, notably DDT, in the cultivation of khat (15). Overall, khat is a central crop in Haramaya and surrounding areas, playing a crucial role in the livelihoods of the people.

Limited studies exist on khat consumption among pregnant women within eastern Ethiopia, specifically the Haramaya District; though a recent study in 2022 found that the prevalence of khat chewing among pregnant women in the Oromia region was 15.5% (16). Among the limited research, evidence suggests that khat use among pregnant women has adverse health outcomes, including but not limited to anemia (17), premature rupture of membranes (18), low birth rate, increased infant mortality, and reduced fetal growth (19). In eastern Ethiopia, there is a growing concern that khat is being used as an appetite suppressant among pregnant women (20), when restrictive dietary behavior has already been established as a predictor of anemia during pregnancy (17). In two studies assessing the magnitude of malnutrition in the Haramaya and Kersa Districts of eastern Ethiopia respectively, one revealed that 19.06% of the 1,731 pregnant women sampled were malnourished, and 23.3% were underweight (21); the second found that the incidence of low birth weight was 28.3% and highly associated with being malnourished and living in rural areas (22). Furthermore, malnutrition, whether associated with severe anemia or another nutrient deficiency, can lead to obstructed labor, premature or low birth weight (23), and postpartum hemorrhage (24)—postpartum hemorrhage being the leading risk factor for maternal death in Africa (25).

Given the cultural importance, high production, and access to khat within the Haramaya District, and limited research regarding the health effects among pregnant and lactating women who chew khat, this study sought to investigate khat utilization among pregnant women. The design and methodology of this study were guided by McLeroy's (26) social-ecological framework, which considers the dynamic interaction among individual, relational, community, and societal influences. This study aims to assess the use of khat and its influencing factors among pregnant women in the Haramaya District of Ethiopia.

## Methods

### Study design and participants

This cross-sectional, explanatory sequential mixed methods study was conducted in the Haramaya District of the Oromia region in eastern Ethiopia using a structured questionnaire and Focus Group Discussions (FGDs). An explanatory sequential design was conducted based on Creswell & Creswell's (27) guidelines that necessitate a logical, structured progression from quantitative to qualitative methods for further exploration. For the questionnaire, pregnant women 18 to 49 years of age residing in the Haramaya District were recruited for the study at the village (kebele) level. Haramaya is part of the Haramaya Health and Demographic Surveillance System (HDSS), which was established in 2018 and is representative of Haramaya District (10). The Haramaya HDSS covers twelve rural kebeles out of 33 kebeles found in Haramaya District wherein eight of the twelve kebeles were randomly selected. Based on the available information within the Haramaya HDSS about the average number of pregnant women in each kebele, we estimated that the required sample size could be obtained from eight of the twelve rural kebeles. The number of pregnant women per kebele was also the basis for the size of the subsample in each kebele. The sample size was estimated using a single population proportion formula with the following assumptions: prevalence of maternal khat chewing during pregnancy (15.9%) with a 95% confidence interval, 10% margin of error, and 10% non-response rate using the following formula, n=[Z2P(1−P)]/d2. The computer-generated lottery method was used for random sampling within each kebele. Pregnant and lactating women over 18 within the eight kebeles were randomly selected and invited to participate in the FGDs. Additionally, representatives from various kebeles, known as the Women's Development Army, were recruited for one FGD.

### Procedures

Structured questionnaires were developed in English and translated into the local language, Afan Oromo. Data collectors were trained enumerators from the Haramaya Health and Demographic Surveillance system who were familiar with the local communities. Pre-testing and training were done prior to data collection to ensure accuracy. All revisions to the instrument were made during the training session and reflected in the findings of this study. Questionnaires were administered to participants by these trained enumerators and assessed sociodemographic information, pregnancy history, and diet, including khat use and factors associated with khat use. All questionnaires were back-translated into English prior to analysis. Data was collected from June 29 to July 7, 2023. The questionnaire was administered verbally in the local language, Afan Oromo, using paper surveys and uploaded to Qualtrics. At the end of each day of data collection, the enumerators and project manager reviewed the questionnaires to identify missing data prior to leaving the kebele. If they found missing data, they went back to the participant prior to leaving the kebele to ensure there was no missing or incomplete data. FGDs expanded on the knowledge, attitudes, and practices of khat in the region and included pregnant or lactating women from two different kebeles. Lactating women participated in the FGDs to provide deeper insights into the findings gathered from the questionnaires among pregnant women. The FGD semi-structured instrument was originally grounded in current literature and collaboration with Haramaya University researchers. While the instrument was reviewed and piloted prior to data collection, it was iteratively revisited to incorporate organic and ongoing discussion points, as analysis and discussion procedures were concurrent. FGDs were conducted in Afan Oromo and recorded; they were later translated and transcribed by a local researcher proficient in the local language and English.

### Ethical considerations

The study was approved by the University of Florida (IRB# 202300400), University of Notre Dame (22-11-7493), and Haramaya University (EFP 2023-3-08) institutional ethical review boards. Written informed consent was obtained from the study participants after explaining the study's purpose, procedure, duration, risks, and benefits.

### Analysis

Quantitative data collected from the questionnaires were analyzed using SPSS v28. The study used descriptive statistics, an independent sample *t*-test, and chi-squared tests to review associations. Further, binary logistic regression using corresponding 95% Confidence Intervals (CI) was used to compute the odds ratios comparing the dependent variable, khat use during pregnancy, separately with each independent variable: age, number of pregnancies, trimester, education, household grows khat, and household sell khat sells khat, to look for significant associations. Multivariate logistic regression was then used to report the adjusted odds ratios for those variables that were considered to have crude odds ratios with a significant association, *p*-value <0.05. Data was inconsistent for the last two questions regarding dietary diversity in the questionnaire and was excluded from analysis.

Two independent researchers conducted qualitative content analysis to examine the qualitative findings from the FGDs (28). Content analysis is a method characterized by its empirical objectivity, employed to examine text, symbols, and signs to furnish quantitative descriptions of their apparent content. Current literature around khat use within Ethiopia and preliminary quantitative data informed the FGD instrument. Transcripts from the focus groups were entered into NVivo 14 to aid in the inductive capturing of salient themes through open coding. Once the two researchers thoroughly reviewed the data, they met until all themes and subthemes were agreed upon, thereby increasing inter-rater reliability (29). Once themes and subthemes were finalized, both researchers were able to operationalize definitions within the final codebook. After finalizing the codebook, the researchers conducted a final round of independent analysis to determine the frequency of each theme and subtheme among FGDs as dictated by Hseih and Shannon (30). Various techniques were employed to ensure the trustworthiness of the data using Lincoln and Guba's (31) four criteria. Credibility was captured through the use of multiple coders to achieve inter-rater reliability (32), transferability through implementing a well-established and frequently cited methodological analysis approach, qualitative content analysis (28, 33). Dependability was established by having the two independent researchers present the final themes and subthemes to the project research team. Finally, confirmability was accomplished by utilizing a qualitative data analysis software, NVivo 14, that provided a systematic way to audit the data (34). This process fostered a more holistic understanding of the impact of khat use on pregnant and lactating women in Haramaya.

## Results

### Quantitative findings

A total of 444 pregnant women ages 18–45 with a median (IQR) age of 25 years (21, 30) completed the questionnaire. The descriptive characteristics of the women are presented in Table 1. Of these women, 63.7% (283) had no formal education and 100% (444) were married and identified as Muslim. The majority of women, 97.3% (432), reported agriculture as a means of livelihood for their household, with 95.3% (423) of households growing khat and 94.4% (419) selling khat. The number of times the participants had been pregnant, including the current pregnancy, ranged from 1 to 5 or more (median 3), with 14% (62) in their first trimester, 36.5% (162) in their second trimester, and 49.5% (220) and in their third trimester.

**Table 1:** Demographics of questionnaire study sample (*n* = 444).

	Total population (*n* = 444)		Pregnant women who do not use Khat (*n* = 147)		Pregnant women who use Khat (*n* = 297)	
	*N*	Percentage %	*N*	Percentage %	*N*	Percentage %
Age, years						
18–20	109	24.5%	74	50.3%	35	11.8%
21–25	123	27.7%	40	27.2%	83	27.9%
26–30	131	29.5%	27	18.4%	104	35%
31–45	81	18.3%	6	4.1%	75	25.3%
Trimester of pregnancy						
1st	62	14.0%	17	11.5%	45	15.2%
2nd	162	36.5%	62	42.2%	100	33.7%
3rd	220	49.5%	68	46.3%	152	51.1%
Number of total pregnancies						
1	80	18.0%	63	42.9%	17	5.7%
2	73	16.4%	31	21.1%	42	14.1%
3	72	16.2%	20	13.6%	52	17.5%
4	66	14.9%	14	9.5%	52	17.5%
5+	153	34.5%	19	12.9%	134	45.1%
Religion						
Muslim	444	100%	147	100%	297	100%
Other	0	0%	0	0%	0	0%
Ethnicity						
Oromo	444	100%	147	100%	297	100%
Other	0	0%	0	0%	0	0%
Marital status						
Married	444	100%	147	100%	297	100%
Other	0	0%	0	0%	0	0%
Educational						
No education	283	63.7%	84	57.1%	199	67%
Education	161	36.3%	63	42.9%	98	33%
Completed grade 1	134	30.2%	54	36.7%	80	26.9%
Read & write only	6	1.4%	2	1.4%	4	1.3%
Completed grade 9	17	3.8%	4	2.7%	13	4.4%
Diploma	4	0.9%	3	2.0%	1	0.3%
Household livelihood						
Agriculture	432	97.3%	138	93.9%	294	99%
Other	12	2.7%	9	6.1%	3	1%
Household grows Khat						
Yes	423	95.3%	135	91.8%	288	97%
No	21	4.7%	12	8.2%	9	3%
Household sells Khat						
Yes	419	94.4%	135	91.8%	284	95.6%
No	25	5.6%	12	8.2%	13	4.4%
Currently smokes						
Yes	5	1.1%	0		5	1.7%
No	439	98.9%	147	100%	292	98.3%
Currently uses alcohol						
Yes	0	0%	0	0%	0	0%
No	444	100%	147	100%	297	100%
Ever used Khat						
Yes	316	71.2%	19	12.9%	297	100%
No	128	28.8%	128	87.1%	0	0%
Currently uses Khat while pregnant						
Yes	297	66.9%				
No	147	33.1%				

Regarding the main outcome variable, 66.9% (297, CI 0.62–0.71), reported currently consuming khat while pregnant, with 71.2% (316) reported yes to ever consuming khat. Almost all women denied smoking 98.9% (439), and all reported no use of alcohol.

Table 2 presents descriptive characteristics of pregnant women who currently use khat. Of the pregnant women who reported currently using khat, the majority use khat daily 72.7% (216) or 3–4 times a week 14.4% (43) The women reported spending an abundant amount of time chewing khat during the day with only 15.2% (45) spending less than an hour or less, 45.1% (134) spending 2–3 h, 23.6% (70) spending 4–5 h, 13.5% (40) spending 5–6 h, and 2.6% (8) spending 6 or more hours per day chewing khat. When asked if their khat consumption had changed during pregnancy, 47.5% (141) said no, 49.2% (146) said they chew less, 2.3% (7) stated they chew more, and 1% (3) reported recent cessation of khat chewing. Half of the women who chew khat, 50.8% (151), started chewing khat more than two years ago, 26.9% (80) started one year ago, 7.1% (21) started less than one year ago, and 15.2% (45) started as a child. In terms of money spent on khat during the past week, more than one-fourth of the women, 26.6% (79) said they did not pay money for khat and had no costs, while the cost for others ranged from less than 140 ETB up to 700 ETB on khat per week, with only 2.3% (7) indicating more than 700 ETB spent. When asked why they consumed khat, the three most important reasons shared were for prayer, to socialize with friends, and to be alert and increase concentration during work.

**Table 2:** Factors associated with pregnant women who currently use khat, based on khat use over the past 3 months (*n* = 297).

	Count (*n* = 297)	Percentage
Started chewing Khat		
As a child	45	15.2%
More than 2 years ago	151	50.8%
1 year ago	80	26.9%
Less than 1 year ago	21	7.1%
Variations in Khat use during pregnancy		
No change	141	47.5%
Chew more	7	2.3%
Chew less	146	49.2%
Stopped	3	1.0%
Single-sitting Khat use quantity		
Less than half bundle (<200 g)	71	23.9%
Half bundle	132	44.4%
Full bundle	20	6.7%
1.5 bundles (>200 g)	36	12.1%
More than 1.5 bundles (>300 g)	38	12.9%
Longest time without using Khat		
1 day or less	151	50.8%
1–6 days	92	31.0%
1 week–1 month	41	13.8%
1 month or more	13	4.4%
Frequency of Khat use in the past 3 months		
Less than once a month	4	1.4%
1–3 days a month	13	4.4%
1–2 days a week	21	7.1%
3–4 days a week	43	14.4%
Daily or almost daily	216	72.7%
Reported amount of time spent chewing Khat throughout the day		
An hour or less	45	15.2%
2–3 h	134	45.1%
4–5 h	70	23.6%
5–6 h	40	13.5%
6 or more hours	8	2.6%
Attempts to reduce Khat use in the past 3 months		
Yes, but not in the past three months	86	28.9%
Yes, in the past three months	24	8.1%
No	187	63.0%
Weekly expenditure on Khat		
No cost	79	26.6%
Less than 140 birr	46	15.5%
140–350 birr	72	24.2%
350–525 birr	56	18.9%
525–700 birr	37	12.5%
More than 700 birr	7	2.3%

Factors significantly associated with pregnant women consuming khat were the number of pregnancies, maternal age, and households that grow khat (Table 3). Specifically, as maternal age increased, the odds of consuming khat increased by 2.03 (CI 0.8–5.13) among the 26–30 age range and 4.95 (CI 1.43–17.22) among the 31–45 age range. Likewise, as the number of pregnancies increased, so did khat consumption. Women in their third pregnancy were 6.51 (CI 2.41–17.62) times, in their fourth pregnancy were 7.52 (CI 2.49–22.72) times, and in their fifth pregnancy were 11.64 (CI 4.11–39.92) times as likely to use khat compared to women in their first pregnancy. Women from households that grow khat were 3.48 (CI 1.34–10.48) times more likely to chew khat than women from households that do not grow khat.

**Table 3:** Factors associated with pregnant women who currently Use khat, logistic regression results (*n* = 444).

Variable	Category	Crude OR	95% CI	*P*-value	Adjusted OR	95% CI	*P*-value
Maternal age	18–20	1			1		
	21–25	4.39	2.53–7.61	<0.001*	1.53	0.70–3.325	0.24
	26–30	8.14	4.54–14.60	<0.001*	2.03	0.8–5.13	0.13
	31–45	6.43	10.49–66.56	<0.001*	4.95	1.43–17.22	0.012*
Number of pregnancies	1	1			1		
	2	4.941	2.42–10.1	<0.001*	3.87	1.68–8.91	<0.001*
	3	10.14	4.79–21.48	<0.001*	6.51	2.41–17.62	<0.001*
	4	13.24	5.95–29.42	<0.001*	7.52	2.49–22.72	<0.001*
	5	26.33	12.82–54.06	<0.001*	11.64	4.11–39.92	<0.001*
Trimester	1	1					
	2	1.16	0.32–1.16	0.61			
	3	1.69	0.45–1.61	0.86			
Education level	No education	1			1		
	Completed grade 1	0.83	0.15–4.64	0.84	0.78	0.1–5.91	0.81
	Read and write only	0.62	0.42–0.95	0.03*	1.23	0.72–2.12	0.45
	Completed grade 9	1.36	0.43–4.28	0.60	2.45	0.69–8.72	0.17
	Diploma	0.14	0.014–1.36	0.09	0.94	0.072–12.24	0.96
Household grows Khat	No	1			1		
	Yes	2.84	1.17–6.91	0.021*	3.48	1.34–10.48	0.017*
Households sell Khat	No	1					
	Yes	1.94	0.86–4.37	0.109			

* Denotes *P*-value <0.05.

### Qualitative findings

Five Focus Group Discussions (FGDs) comprising 40 women total with eight women per FGD were conducted throughout Haramaya District. The sample of women varied in age from 18 to 60, with a mean age of 30.9 and an average of 4.4 children (Table 4). Of those sampled, 70% (28) had no formal education, with the highest educational attainment being grade 10. The FGDs lasted between 45 min to 1 h and 10 min. Researchers used content analysis to analyze the FGD transcripts, resulting in the discovery of five themes: Economic Livelihood, Maternal Significance, Medicinal Implications of Khat, Pesticide Use, and Social and Cultural Applications. Findings related to each of these themes were further elucidated through various subthemes and operationalized within Table 5. Each participant was provided a code based on their location and number within the FGD.

**Table 4:** Focus group discussion demographic characteristics.

Demographic characteristics	Pregnant women (*n* = 16)	Lactating women (*n* = 16)	Women's development army (*n* = 8)
	*N* (%)	*N* (%)	*N* (%)
Age			
18–25	6 (37.5)	8 (50)	1 (12.5)
26–35	5 (31.3)	6 (37.5)	2 (25)
36–45	5 (31.3)	2 (12.5)	3 (37.5)
46+	0 (0)	0 (0)	2 (25)
Education status			
No formal education	11 (68.75)	12 (75)	5 (62.5)
Grades 1–5	4 (25)	2 (12.5)	1 (12.5)
Grades 6–10	1 (6.25)	2 (12.5)	2 (25)
Ethnicity			
Oromo	16 (100)	16 (100)	8 (100)
Religion			
Muslim	16 (100)	16 (100)	8 (100)
Number of pregnancies			
1–5	8 (50)	9 (56.25)	1 (12.5)
6–10	7 (43.75)	7 (43.75)	7 (87.5)
More than 10	1 (6.25)	0 (0)	0 (0)
Years chewing Khat			
None	4 (25)	2 (12.5)	1 (12.5)
1–5 years	5 (31.3)	4 (25)	2 (25)
6–10 years	3 (18.75)	3 (18.75)	2 (25)
11–15 years	2 (12.5)	3 (18.75)	0 (0)
15–20 years	2 (12.5)	3 (18.75)	0 (0)
More than 20 years	0 (0)	1 (6.25)	3 (37.5)

**Table 5:** Focus group discussion codebook.

**Economic Livelihood**: Discussions on how Khat is used to generate income for a household through its sale as well as its use. ○ **Khat as a source of income**: Any mentioning of Khat as a source of income for a household or as a method of purchasing goods or services. This also includes discussions on the price of Khat as it pertains to household income. ○ **Motivation to work**: Comments made regarding the use of Khat in order to complete work/tasks and its different applications among men and women. **Maternal Significance**: Conversations concerning how Khat use impacts pregnant women, the child's gestation, and the postpartum period. ○ **Concerns with Khat use and breast milk quality/quantity**: Claims made addressing whether Khat use influences breast milk quality/quantity, and if so, what those impacts are. ○ **Disputes over Khat's benefit among pregnant and/or lactating women**: Deliberations over the advantageousness of Khat for pregnant women and lactating mothers. ○ **Disputes over Khat's safety among pregnant and/or lactating women**: Deliberations over the risks, or lack thereof, of Khat for pregnant women and lactating mothers. **Medicinal Implications of Khat**: Explanations of the numerous health-related consequences of Khat use ○ **Addictive behaviors**: Either explicit or implicit references to the addictive nature of Khat and how this addiction impacts the individual. ○ **Appetite suppressant**: Comments made explaining how Khat is both purposefully as well as inadvertently used to suppress appetite, and the consequences of this appetite suppression. ○ **Health status when using Khat**: Clarifications as to when one should chew Khat based on their health status. ○ **Khat as a stimulant**: Expressions made highlighting how individuals use and rely on Khat as a stimulant to complete activities. ○ **Physical manifestations**: Descriptions of both the positive and negative effects of Khat use on one's physical health. **Pesticide Use**: Discussions on the application of pesticides during Khat cultivation and the subsequent measures taken to avoid pesticide ingestion. ○ **Distinguishing between chemicals and organic Khat**: Personal anecdotes made describing how one can delineate between pesticide-treated Khat and organic Khat. ○ **Gender differences and dynamics**: Explicit discrepancies made by participants on the basis of one's gender regarding the impacts of pesticide practices. ○ **Perceptions of washing and cleansing Khat**: Practices enacted to lessen the potency of the pesticides through washing and cleansing and the beliefs justifying these practices. **Social and Cultural Applications**: Expectations of Khat use in formal and informal social and cultural contexts. ○ **Casual gatherings**: Any mention of Khat used in commonplace gatherings on a regular basis with neighbors and/or friends. ○ **Ceremonial use**: Any discussion as to how Khat is used in larger events/ceremonies and expectations of its use specific to these events/ceremonies. **Prayer**: An objective expression of the use of Khat in prayer or reverence to God.

#### Economic livelihood

Responses from FGD participants were coded as economic livelihood if they illustrated how khat was used to generate income for a household, through both its sale locally or exported, as well as its use. All of the FGDs discussed how khat impacts their financial situation (*N* = 5).

Pregnant Participant F4: “People who do not have khat do not have a good livelihood. Someone who has a khat farm and those who do not have a khat farm do not have the same livelihood. People say this person has a khat farm and this person has no khat farm. Those who do not have a khat farm may not get a loan. They cannot borrow from people. You can easily get a loan if you have a khat farm. People see your khat farm quantity before they lend you money. They say “his khat farm is not good and he may not pay back his loan”. Therefore khat is our livelihood. You will not be accepted in the community if you don't have a khat farm”.

These discussions were further broken down into two subthemes: khat as a source of income and motivation to work. From the FGDs that mentioned khat as an income source (*N* = 3), they highlighted how khat sales support the family, generating income to purchase other goods and services.

Lactating Participant IO3: “Our earnings are mainly from khat. We cut it and sell it to survive. We sell it to buy food for our children. We feed our family using the money we earn from selling our khat. We also use it to cover our health expenses. Someone who has no khat may find it difficult to survive. It is our currency. But the price nowadays is very low”.

The FGDs commenting on khat as a motivation to work (*N* = 5) emphasized how the substance was seen as vital in completing work and tasks.

Pregnant Participant F2: “Both men and women use it in the early morning to be active for work. If we don't use it we feel weak. We need to chew small amounts to get activated for our daily activity. Our livelihood is completely dependent on it.”

#### Maternal significance

Remarks coded under maternal significance addressed how khat use impacts pregnant women, the pregnancy, and the postpartum period. Each of the FGDs spoke on topics of maternal significance (*N* = 5).

Women's Development Army F6: “I advise pregnant mothers that khat use during pregnancy has no benefit to her health and the health of your baby. I advise her that if she becomes stimulated she will engage in heavy work. She may fall while engaging in heavy duties. It can also cause changes in the position of the fetus. This can increase early labor. Her leg may swell. Some others also fall while engaging in heavy works.”

Conversations were being critically examined in the subthemes around concerns with khat use and breast milk quality/quantity; disputes over khat's benefit among pregnant and/or lactating women; and disputes over khat's safety among pregnant and/or lactating women. Every FGD engaged in these subthemes (*N* = 5), with the latter two addressing the efficacy and risks of using khat for mothers, and the former emphasizing how khat may or may not influence the mother's breast milk. One woman describes her feelings of concern with khat use and breast milk quality/quantity:

Pregnant Participant F1: “Using khat while taking care of small babies has no benefit. This is because there are times when you exclusively breastfeed your baby. At this time, your baby gets what you eat. Using khat while breastfeeding decreases the milk quantity and the child may not get adequate breast milk. The baby may be exposed to malnutrition. His body may swell. Therefore, I personally believe using khat while breastfeeding is not important. It has no benefit to both the mother and the baby other than causing health problems”.

While most participants believe khat to be harmful or have no positive effects, some participants acknowledged how khat use is essential despite being pregnant or breastfeeding. The subtheme, disputes over khat's benefit among pregnant and/or lactating women, is illustrated here by one mother:

Pregnant Participant F8: “Yes, pregnant and lactating mothers also use khat. It has a benefit for me as well as a side effect on me. It has a benefit for me because after lunch I have to use khat because I have already developed a dependency. I mean, I will not be active in my work unless I use khat. When I use it I will feel strong and stimulated to do my job. But it will affect my health if I use [it] too much. It causes light-headedness when I stand up”.

The subtheme, disputes over khat's safety among pregnant and/or lactating women, illustrated how some believed there were deleterious effects. In contrast, others stated there were no health issues related to khat use.

Pregnant Participant F3: “There is no problem we face because of using khat. We have other health problems due to our pregnancy, not because of using khat. Khat has no effect on people. We even get better after we use khat if we have any health problems. We get better when we use khat. Khat gives you strength”.

#### Medicinal implications of Khat

Claims made by FGD participants regarding the numerous health-related consequences of khat use were coded as medicinal implications of khat and were captured across all FGDs.

To expand on specific trends discovered through the FGD responses, five subthemes were generated. These subthemes, addictive behaviors (*N* = 5), appetite suppressant (*N* = 4), health status when using khat (*N* = 4), khat as a stimulant (*N* = 5), and physical manifestations (*N* = 5), each involved deliberation as to how khat use impacted their physical well-being. The subtheme, addictive behaviors, describes the dependence many spoke of around khat. One woman specifically stated:

Lactating Participant F2: “Many people use khat because of dependence or addiction. They will sleep if they don't get khat. They will not work if they don't use it. The parents may beat them to wake up and go to work. They have to search for khat anywhere so that they will use it to be active for work”.

The subtheme, appetite suppressant, was relevant for many women as they discussed their khat use behaviors concerning the availability of food. More specifically, women discussed how an appetite suppressant was used intentionally or unintentionally depending on how much food was available for the household.

Pregnant Participant F7: “We give the available food to our children and older adults use khat when they get hungry. We don't care about hunger when we use khat”.

Within the subtheme, health status when using khat, women agreed across FGDs that only healthy people should chew khat, not those who feel ill or considered unhealthy. Being unhealthy was often described as having a nutritional deficiency, but otherwise was not elaborated on.

Pregnant Participant F3: “It is not good to chew khat when you are unhealthy. But it has no effect when you are healthy. I mean if you are healthy or have no health problems. But if you use it when you are healthy it improves your health. You become stronger”.

Khat was discussed often as a stimulant leading to the manifestation of the subtheme, khat as a stimulant, which was used to help improve motivation and energy for daily functions.

Lactating Participant IO7: “Khat is used as a stimulant and you will search for work if you are stimulated. We are unable to do our household activities unless we use khat”.

The final subtheme, physical manifestations, were described during the FGDs as stomach aches, nausea, cramping, and other abdominal pain, among others.

Lactating Participant F5: “But many pregnant mothers don't like to use khat because it causes belching. It causes gastric upset so they immediately stop using it. Some also decrease the amount that they use. It may worsen the nausea and vomiting of pregnancy. It may also cause sleeping disorders for mothers if they get stimulated too much”.

#### Pesticide use

Participants frequently spoke about the application of pesticides during khat cultivation as well as the subsequent measures taken to avoid pesticide ingestion, with these descriptions being coded as pesticide use (*N* = 4). To further exemplify the beliefs and behaviors related to pesticides and khat, the following subthemes were identified: distinguishing between chemicals and organic khat (*N* = 4), gender differences and dynamics (*N* = 2), and perceptions of washing and cleansing khat (*N* = 4). With each subtheme, the participants provided personal anecdotes detailing how pesticides are harmful, especially for women and pregnant mothers, and mitigation strategies that they use, either cleaning the khat or avoiding pesticide-treated khat altogether. Within the subtheme, distinguishing between chemicals and organic khat, a participant went so far as to say pesticide-treated khat can cause abortion among other lethal outcomes.

Lactating Participant IO8: “If you chew with pesticide-treated khat it will tear your abdominal organs. It will bring a death angel on you. It will cause abortion. Your breastfeeding baby will also feel unhealthy”.

Interestingly, within the subtheme, gender differences and dynamics, women explained how men and children apply pesticides to their own khat, not women. However, women are permitted to sell khat locally at market.

Lactating Participant F1: “No, women cannot apply the pesticides. Mostly our husband applies the pesticide, but in the absence of our husband, our older children will apply the pesticide. Women ask their male neighbors to apply pesticides on their khat farm”.

Due to the widespread recognition of pesticide presence, women also openly described various beliefs around how to properly wash khat to remove pesticides. A participant describes the subtheme, perceptions of washing and cleansing khat, here:

Lactating Participant F7: “It depends on the type of pesticide. People use DDT-treated khat anytime they want. But we wait for at least one month before using *hatattam* [methomyl/90 sp and aragonite/90 sp] treated khat. No one goes into the khat farm once it is treated with *hatattam* until one month has passed. It smells so powerful no one can get close to it”.

Another woman describes using lemon and milk to wash away the pesticides.

Pregnant Participant F6: “We use lemon and milk in case we fear poisoning. Urban people use this method, but farmers do not mostly buy khat from the market”.

#### Social and cultural applications

Khat held social and cultural significance among the FGD participants, with the expectation of its use coded as social and cultural applications (*N* = 5); these were further elucidated by the context in which khat was used and/or how it was used. In the subthemes of casual gatherings (*N* = 4), ceremonial use (*N* = 4), and prayer (*N* = 4), participants emphasized that khat has significance beyond its perceived physical and monetary value, bringing people together and holding significant cultural value. The subtheme, cultural gatherings, depicts any mention of khat used in commonplace gatherings on a regular basis with neighbors and/or friends.

Lactating Participant IO2: “Pregnant and lactating mothers occasionally gather with their neighbors and use khat for socialization. We prepare hoja [coffee] and enjoy our khat together. We then depart to our work. Pregnant mothers also use khat with people. They don't usually do it for a long time, they just sit for a few minutes and return back to their work”.

The subtheme, ceremonial use, was defined as any discussion as to how khat is used in larger events or ceremonies and expectations of its use specific to these events/ceremonies.

Lactating Participation F7: “We use khat with other people during *hafosha* [social gathering such as a wedding ceremony, mourning/grievance, and holidays] like during wedding ceremonies. They bring us hoja and khat. We sit with the people to use the khat until the wedding ceremony ends. We also use it when we go to Gumata [traditional repentance for doing harm to people]. We sit with both families as a traditional rule, have khat and drinks and pay our repentance. We use khat from our farmland or we buy it from the market with other people. But pregnant and lactating mothers do not sit for long to chew khat with people. They just sat for a few moments and departed with the other people”.

In the final subtheme, prayer, khat was described as an objective expression in prayer or reverence to God.

Lactating Participant IO1: “We bring special khat and pray to God to bless our days. We pick the leaf toward God and pray to him to solve our problems. We use it for prayer”.

## Discussion

The reported prevalence of pregnant women surveyed within this study who indicated chewing khat was 66.9%. This figure drastically exceeds the prevalence of khat use among pregnant women (19.6%) throughout Ethiopia in 2021 (35). However, there are conflicting studies in the Jimma Zone of Ethiopia with variations in khat use among pregnant women, ranging from 19% (36) to 65.8% (37). One noticeable difference is that both studies from the Jimma Zone were health facility-based, while ours was a community-based study. Women who attend antenatal care (ANC) visits are less likely to engage in risky behaviors and substance and are more likely to receive awareness creation on the potential adverse effects of khat consumption during pregnancy (36). When examining the Haramaya District in Ethiopia and focusing specifically on community-acquired data, the most recent reported prevalence of khat use among pregnant women was 15.5% in 2022 (16). Despite the Jimma findings being unclear, several studies have postulated that a driver of high khat use among a population is its accessibility, specifically whether they are producers of khat (36, 38); this is especially true within this study's findings where 95.3% of pregnant women reported growing and 94.5% reported selling khat. The age, education level, marital status, and partner use findings were congruent with other studies (36, 39); however, tobacco and alcohol use were significantly lower than in other regions of Ethiopia (35). This variation could be attributed to the sociocultural differences within study populations, given that other studies from the same population had similar findings (16). Our study population was entirely composed of communities of the Muslim faith, in which alcohol is considered *haram* or forbidden.

The prevalence of pregnant women who reported daily khat use was 72.7%, with almost half (45.1%) spending between 2 and 3 h per day and 23.6% spending 4–5 h per day chewing khat. Nakajima et al. (36) found that, of the women who chewed khat, substantially fewer pregnant women from the Jimma Zone chewed khat daily (13%). Additionally, pregnant and lactating women within the Focus Group Discussions (FGDs) described khat as a dependence or an addiction among the population. Several women debated whether khat has any health benefits, especially during pregnancy. At the same time, some argued that “We get better when we [pregnant women] use khat. Khat gives you strength”. Others postulated that khat use during pregnancy may cause harm to the mother and infant. Despite this argument, the need to use khat for energy or because of being dependent often outweighed the potential for harm. Other similar studies described chronic khat use as meeting the Diagnostic and Statistical Manual of Mental Disorders (DSM) criteria for a substance abuse disorder (40). According to Kalix (6), khat's quick onset of effects can lead to a greater likelihood of users developing a stronger dependence than amphetamine. Furthermore, while other studies reported a stigma around khat use among women (41), this study's findings indicated a more substantial stigma around addiction than khat use. Since khat is addictive, the two cannot be discussed in isolation, however, they are presented this way within this community. This discrepancy could be a potential barrier to khat reduction or mitigation. This difference may be due to the perceived normalization of khat use among the general population in the eastern Oromia Region and the economic reliance on khat as a cash crop (4). Khat is vital at the household level for income stability as a cash crop and for endurance, it presents as a stimulant for energy to perform daily activities. As a result, this reliance extends beyond addiction; it establishes an interminable pattern of dependence in which an individual's existence becomes wholly centered on khat, serving not only as a source of income but also as the path to securing that income.

Interestingly, the FGDs with pregnant and lactating women detailed reasons for limiting pesticide use within their khat farms and avoiding “chemically” treated khat for household consumption. Several pregnant and lactating women described most chemicals used to treat khat as noxious and causing health-related issues, ranging from gastrointestinal illness to headache to abortion or even death. According to Oyugi and colleagues (42), smallholder khat farmers have been found to employ various chemicals, including dichlorodiphenyltrichloroethane (DDT), wuhagare (also spelled wuhagar‘ or wuha agar), sevin, malathion, actellic, diazinon and organophosphate pesticides. Unlike other studies, participants in this study described methomyl/90 sp. and aragonite/90 sp., with the Afan Oromo word *hatattam* used for both insecticides. Among both FGD sites, *hatattam* was described as “the death angel” because it can kill the pregnant mother, cause abortion, and even kill a grown man. Methomyl, classified as an N-methyl carbamate insecticide, is primarily used to manage insects and pests in foliage and soil. Exposure to methomyl through ingestion, eye contact, or inhalation is associated with a high to moderate level of toxicity. Moreover, methomyl acts as a cholinesterase inhibitor in humans when exposed through the oral, dermal, or inhalation route. Consequently, if an individual encounters methomyl through ingestion, skin contact, or inhalation, it can lead to an overstimulation of the nervous system, resulting in symptoms such as nausea, dizziness, confusion, and, in cases of exceptionally high exposures (e.g., accidents or major spills), respiratory paralysis and potentially fatal outcomes (43).

Based on the FGD descriptions of khat cleaning practices and separating khat based on chemical vs. organic farming practices within the plot of land, it is suspected that the exposure to methomyl, and the other chemicals mentioned above, was high. For example, the pregnant women described the following tactics to mitigate the adverse effects: using lemon and milk to decrease poisoning, allowing the rain to wash away the chemicals on the khat, prescribing a determined amount of time to wait based on the chemical used, and having a small plot of their land used exclusively for organic growing. While these practices may be perceived as reducing exposure, it is likely that through residual spraying, absence of personal protective equipment, contamination of groundwater and soil, or other exposure pathways, the risk remains high. In a recent study to capture contemporary knowledge around pesticide-related risks among Ethiopians (44), it was found that farm workers have poor knowledge and understanding of pesticide use, handling, and management; in some cases, they engaged in incompatible mixing of pesticides, over spraying crops, and improper disposal. With the ubiquitous use of pesticides and fertilizers to improve food and crop production, exposure to toxic chemicals through various pathways increases the risk to consumer health. Adverse health effects associated with the chemicals used on khat have already been documented (42), with health diseases ranging from a spectrum of different cancers from DDT, liver damage from heavy metals and pesticides, hypertension, and a deluge of other diseases. Pregnant and lactating women are especially at risk of passing chemicals, such as DDT, through breast milk or affecting the neurodevelopment of a fetus *in utero* through transplacental transfer (45). Interestingly, when asked whether participants in one of the FGDs thought khat specifically caused cancer, there was a strong belief that it did not. However, when asked if the chemicals used on khat were cancer-causing, there was a shared belief that they did.

One of the strengths of this study was the unprecedented prevalence of khat consumption among pregnant women captured within the eastern Oromia Region. To control for selection bias, researchers used well-established and trusted avenues for recruitment through the Haramaya HDSS, randomized kebeles and households were targeted, and a representative sample of the population based on the Haramaya HDSS most recent data was captured (46). Moreover, there appeared to be less concern around the stigma of reporting khat use while pregnant compared to other studies. This may be due to the rising normalization of khat use among women and children, specifically within Haramaya. Another strength is the novel finding where it seems that individuals are more concerned with the effects of and exposure to pesticides, than khat consumption. This perspective is critical within harm reduction approaches to provide services that individuals believe to be necessary to foster positive change. Identified weaknesses include the use of a cross-sectional design, the lack of external validity given the pervasive nature of khat in this region, and that nutrition and health outcomes were not assessed. Moreover, because we do not know the exact prevalence of khat production and selling within Haramaya's HDSS data, we cannot say with certainty the sample presented is representative of the district. Furthermore, while mixed methods research improves rigor, there is still an immediate need for long-term, longitudinal studies on the adverse health outcomes among khat users, specifically pregnant and lactating women. Since khat is a known stimulant and appetite suppressant, longitudinal studies assessing how this may contribute to low birth weight and other adverse maternal and birth outcomes are necessary for future research. Following this can include interventions to mitigate khat consumption's adverse health and birth outcomes during vulnerable times such as pregnancy and breastfeeding among practitioners and interventionists in the region. The reported chemicals identified in this study have well-documented histories of adverse health effects, including cancer, raising concerns for pregnant and lactating women passing toxins to their infants. Accordingly, these findings indicate a need for further interventions to improve agricultural practices around pesticide and fertilizer use among khat farmers and their households. This may include but is not limited to more restrictive policies and policy enforcement around illegal pesticides as well as comprehensive education around pesticide use and its health effects if used improperly.

## Data Availability

The raw data supporting the conclusions of this article will be made available by the authors, without undue reservation.
